# No birth-associated maternal mortality in Japanese macaques (*Macaca fuscata*) despite giving birth to large-headed neonates

**DOI:** 10.1073/pnas.2316189121

**Published:** 2024-10-07

**Authors:** Katharina E. Pink, Barbara Fischer, Michael A. Huffman, Takako Miyabe-Nishiwaki, Naoko Suda-Hashimoto, Akihisa Kaneko, Bernard Wallner, Lena S. Pflüger

**Affiliations:** ^a^Department of Obstetrics and Gynecology, Division of Obstetrics and Feto-Maternal Medicine, Medical University of Vienna, Vienna A-1090, Austria; ^b^Department of Evolutionary Anthropology, University of Vienna, Vienna A-1030, Austria; ^c^Department of Evolutionary Biology, Unit for Theoretical Biology, University of Vienna, Vienna A-1030, Austria; ^d^Konrad Lorenz Institute for Evolution and Cognition Research, Klosterneuburg A-3400, Austria; ^e^Wildlife Research Center, Kyoto University, Inuyama 606-8501, Japan; ^f^Center for the Evolutionary Origins of Human Behavior, Kyoto University, Inuyama 484-8506, Japan; ^g^Department of Behavioral and Cognitive Biology, University of Vienna, Vienna A-1030, Austria; ^h^Austrian Research Center for Primatology, Ossiach A-9570, Austria

**Keywords:** maternal mortality, primates, evolution of birth, feto-pelvic disproportion

## Abstract

Human fetuses at term are large relative to the dimensions of the maternal birth canal, implying that their birth can be associated with difficulties. The tight passage through the human birth canal can lead to devastating outcomes if birth becomes obstructed, including maternal and fetal death. Although macaques have to accommodate similarly large fetuses, relative to their maternal birth canals, it was not known whether macaque mothers face birth difficulties similar to humans. Based on 27 y of demographic data from a semi-free-ranging, closely monitored population of Japanese macaques (*Macaca fuscata*), we found no birth-associated mortality in macaques. This differs from the situation in many human populations. We suggest three nonmutually exclusive hypotheses to explain these observations. i) The macaque fetal skull is similarly flexible as the human fetal skull. ii) The macaque pelvis and connective tissue show greater flexibility during birth. iii) The interplay between macaque pelvic shape and birth dynamics is smoother and incurs fewer complications than in humans.

## Significance

A tight fit between the neonatal head and maternal pelvic dimensions is often associated with an increased maternal mortality risk in humans and some other primate species. Japanese macaques show a human-like tight feto-pelvic fit. Based on 27 y of data from a continuously monitored semi-free-ranging group of Japanese macaques, we found no birth-associated maternal mortality in macaques, which differs from the situation in many human populations.

**Fig. 1.**
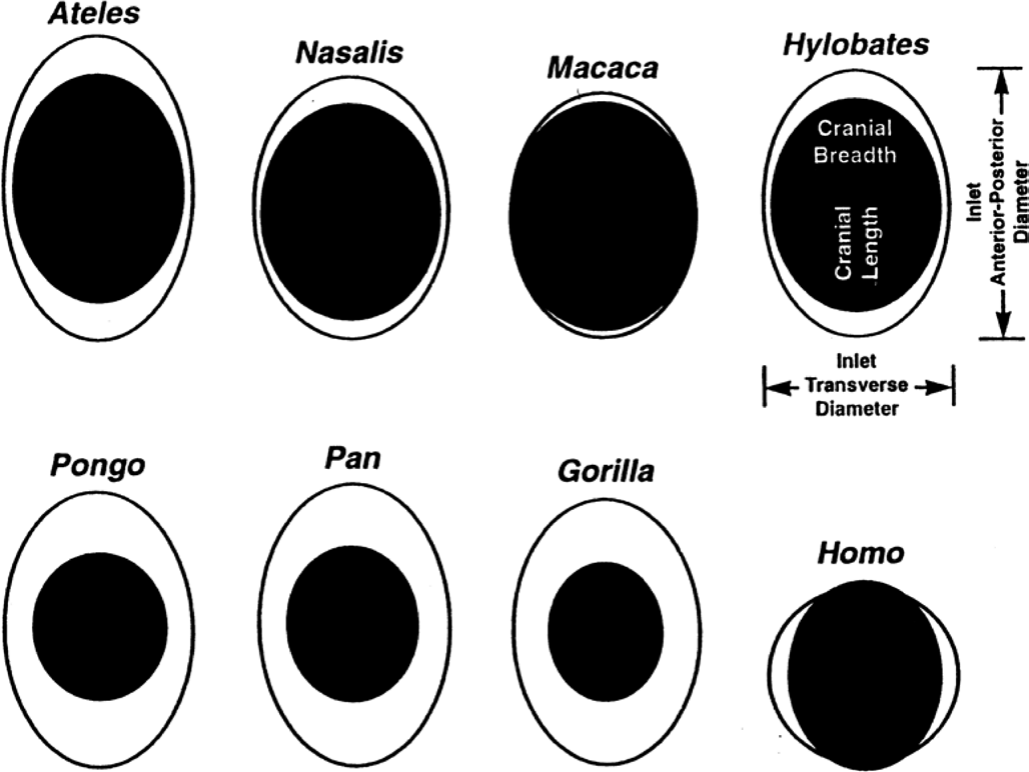
Comparison of the size of the fetal head (black oval) relative to the size of the maternal birth canal (pelvic inlet, white oval) at the time of birth for humans (*Homo*), gorillas (*Gorilla*), chimpanzees (*Pan*), orangutans (*Pongo*), spider monkeys (*Ateles*), long-nosed monkeys (*Nasalis*), macaques (*Macaca*), and gibbons (*Hylobates*). The birth canals are scaled to the same mediolateral width for comparison. From Rosenberg and Trevathan (2) After Schultz (3) and Leutenegger (4).

Birth is typically uncomplicated for mammals that give birth to tiny neonates relative to the size of the maternal birth canal, but more difficult for species with relatively large fetuses (1), especially for certain primates including humans and macaques (Fig. 1). Because cephalopelvic proportions are similar in humans and macaques, one would assume that macaques face similarly difficult births as humans with comparable birth-related morbidities and mortality (Table 1).

**Table 1.** Cephalopelvic dimensions

		Publ.	Population	Method	N	Measure	Values [mm]
*Macaca fuscata*	Pelvis	(5)		CT	4	inlet AP	range: 66.7 to 75.0
						inlet ML	range: 56.8 to 64.4
	Fetal head	(6)	male neonates	after birth	214	head length	mean = 70.1
						head breadth	mean = 54.6
			female neonates	after birth	163	head length	mean = 67.0
						head breadth	mean = 52.2
		(5)		CT	4	fetal skull length	range: 62.2 to 68.5
						fetal skull width	range: 51.1 to 55.6
*Macaca mulatta*	Pelvis	(3)		skeletal material	41	inlet AP	mean = 67.7
						inlet ML	mean = 50.9
		(7)		CT	12	inlet DV	range: 63.9 to 72.2
						inlet ML	range: 48.9 to 57.9
	Fetal head	(3)		skeletal material	28	fetal skull length	mean = 66.3
						fetal skull width	mean = 50.7
		(7)		CT	12	fetal skull AP diameter	range: 60.5 to 72.3
						fetal skull ML diameter	range: 45.5 to 52.1
		(8)		ultrasound	54	biparietal diameter	mean = 50.2
*Homo sapiens*	Pelvis	(9)	UK	radiographs	500	obstetric true conjugate diameter	mean = 118.3 SD = 10
						transverse diameter at brim (inlet)	mean = 130.3 SD = 7
		(10)	Switzerland	MRI	781	obstetric conjugate	mean = 116.5 SD = 10.6
						transverse diameter	mean = 126 SD = 10
		(11)	Germany	CT	25	obstetric conjugate	mean = 118 SD = 2
						transverse inlet	mean = 129 SD = 1
	Fetal head	(12)	USA	after birth	38	biparietal diameter	mean = 91.8
		(13)	UK, male neonates	after birth	106	head length	mean = 120.7 SD = 4.3
						head breadth	mean = 96.0 SD = 3.1
			UK, female neonates	after birth	106	head length	mean = 118.4 SD = 4.6
						head breadth	mean = 94.9 SD = 3.7
		(14)	Sweden	ultrasound	19	occipito-frontal diameter	mean = 107.2 SD = 3.1
						biparietal diameter	mean = 94.9 SD = 3.6
		(15)	International	ultrasound	4,607	occipito-frontal diameter	mean = 115.8 SD = 6.2
						biparietal diameter	mean = 94.9 SD = 3.9
		(16)	India, male neonates	after birth	29	head length	mean = 100.3 SD = 7.4
						head width	mean = 91.7 SD = 7.9
			India, female neonates	after birth	16	head length	mean = 100.5 SD = 5.2
						head width	mean = 88.9 SD = 5.7

Diameters of the maternal pelvic inlet and the fetal head at birth in macaques and humans. The data are compiled from published studies on humans and macaques. For fetal head size at birth, we included head length (or occipito-frontal diameter) and head width (or biparietal diameter). Note that some measurements of the fetal head were taken after birth and might therefore be affected by molding of the skull. For the pelvis, the anteroposterior diameter of the pelvic inlet (inlet AP, also called dorsoventral diameter of the inlet or obstetric conjugate), the mediolateral diameter of the pelvic inlet (inlet ML or transverse diameter) and dorsoventral diameter of the pelvic inlet (inlet DV) are included. To derive cephalopelvic proportions for macaques and humans, we calculated the ratios of the corresponding diameters of the fetal head and maternal pelvic inlet for both species. For macaques, the used values stem from Morimoto et al. (5) and are based on mother-fetus dyads. For humans, we used the consensus values from the INTERGROWTH study of the WHO (15) for fetal head size at full gestation together with pelvic inlet diameters from Keller et al. (10). Note that the longest diameter of the head is aligned with the mediolateral diameter of the pelvic inlet in humans, while it is aligned with the anteroposterior diameter in macaques.

Cephalopelvic proportions for macaques: mean cpp_length = 0.92 for fetal skull length/inlet AP diameter; mean cpp_width =0.90 for fetal skull width/inlet ML diameter.

Cephalopelvic proportions for humans: cpp_width = 94.9/116.5 = 0.81 for fetal skull width/inlet AP diameter; cpp_length= 115.8/126 = 0.92 for fetal skull length/inlet ML diameter.

Maternal mortality, as defined by the WHO (17, 18), describes female deaths while pregnant or within 42 d of termination of the pregnancy, from any cause related to the pregnancy or birth. In 2020, human maternal mortality ranged from 3 maternal deaths per 100,000 live births in Finland to 1,223 maternal deaths per 100,000 live births in South Sudan with its poor medical infrastructure, malnutrition, and conflicts (19). The global decline in maternal mortality is predominantly caused by the widespread availability of high-quality midwifery as well as the availability of antibiotics in postnatal care (20–22).

The leading causes that were responsible for more than half of maternal deaths worldwide between 2003 and 2009 were obstetric hemorrhage (27.1%), hypertensive disorders of pregnancy (pre-eclampsia and eclampsia, 14.0%), and sepsis (10.7%), see ref. 23. In general, the frequency of human natural maternal mortality in settings with no availability of effective medical intervention, including well-trained midwives, is estimated at around 1.5% (24). When comparing maternal mortality across populations, it is important to apply the same definition, as often, estimates of maternal mortality in historical human populations are based on registry data, which does, for example, not reflect deaths during pregnancy. Comparable historical estimates based on a similar definition of maternal mortality as the WHO defines it range from 0.8% in Sweden (25) up to 1.57 to 2.1% in England (22, 26, 27). These rates are similar to estimates for maternal mortality in current hunter-gatherer societies ranging from 1.3% (Hiwi, see 28) to 3.52% (Agta of the Philippines, see 29).

The mammalian pelvis must serve two opposing purposes: It defines the birth canal and at the same time has to enable locomotion (3). Because fetuses are relatively large in humans and macaques, complications such as obstructed labor can occur due to a mismatch between fetal size and birth canal dimensions (3, 30). From an evolutionary point of view, cephalopelvic disproportion and obstructed labor associated with increased maternal and infant mortality as well as morbidity should be selected against. Indeed, Fischer and Mitteroecker (31) have shown that human females, but not males, have evolved a link between head size and pelvis shape to ameliorate this tight fit. However, not only humans but also small- and medium-sized primates such as squirrel monkeys (32) and macaques (3, 7) face a tight cephalo-pelvic fit at birth (Fig. 1) and obstructed labor can occur (33, 34). Kawada et al. (7) demonstrated that a relationship similar to that described by Fischer and Mitteroecker (31) for humans exists in rhesus macaques (*Macaca mulatta*) between maternal pelvic form and fetal head size. Their study suggests that maternal pelvis shape, stature, and neonatal head size also coevolved in other primates, not just in humans, to ameliorate difficulties in the birth process. Although pelvic and fetal dimensions would suggest that birth is similarly difficult in humans and macaques (Fig. 1 and Table 1), it is an open question if this is indeed the case.

Quantitative descriptions of the birth process in semifree and free-ranging nonhuman primates are rare, mostly because birth occurs during the inactive phase of the day (35) and therefore cannot be easily observed. In (semi-) free-ranging Japanese macaques (*Macaca fuscata*), a seasonally breeding non-human primate species, very few births have been described in the literature (36–39). Unlike human birth, where the active phase of the first stage of labor was estimated to last on average 7.7 h for nullipara and 5.6 h for multipara (40), Japanese macaques’ birth seems to be much quicker, lasting from 15 min to three hours. The infants are mostly born head first, either in occiput posterior [facing to the front, (36)] or in occiput anterior position [facing backward, (41)]. Prolonged labor was usually associated with an abnormal fetal position (breech position) or stillbirth (39). Like in humans, multiparous females usually have easier and quicker births, and infant mortality risk is lower in primiparous females (36). Moreover, recent research has shown that Japanese macaques exhibit continuous development of pelvic shape during adulthood leading to obstetrically more favorable pelvic dimensions at advanced ages (5).

Despite these observations and although the tight fit through the pelvic inlet of the birth canal (Fig. 1) seems to be similar in humans and macaques, it is unknown whether birth in macaques is associated with a maternal mortality risk similar to humans. Our study aimed to investigate whether Japanese macaques encounter mortality and morbidity due to obstructed labor and its associated complications, or whether evolution has found a way to ease birth in macaques despite their tight cephalopelvic fit.

We investigated the occurrence of maternal mortality in a provisioned semi-free-ranging population of Japanese macaques from Affenberg Landskron (Carinthia, Austria). The Affenberg population was established in August 1996 from translocated individuals of the free-ranging Minoo H group (Osaka, Japan). From 1997 onward, births and deaths within the population were recorded continuously. This population is ideal to investigate whether birth in macaques is associated with an increased maternal mortality risk for the following reasons: i) the availability of 27 y (1997–2023) of complete demographic data (births, deaths, and age) of all group members; ii) all females produced at least one offspring before being sterilized; iii) the animals lived under semi-free-ranging conditions in a naturally forested enclosure; iv) the caretakers kept track of the health conditions of all individuals. Births occurred without the attendance of caretakers, but birth events and newborns were quickly noticed; and v) no prophylactic treatments were performed to prevent birth complications or pregnancy-related difficulties. To answer our research question, we analyzed the survival time for each female between the last birth and the date of death in the Affenberg population. We also analyzed the distribution of female deaths over time and whether deaths were associated with the birth season (March–July).

## Results

### General Mortality.

During the observation period, a total of 139 deaths (66 females, 67 males, six of unknown sex) were recorded. Out of the 66 female deaths, the full date of death (day, month, year) was available for 60 individuals. For 63 females, the month and year of death were available. The female mean age at death of all individuals above one year of age was 16.9 y (SD = 7.98 y, N = 47).

### General Births.

**Fig. 2.**
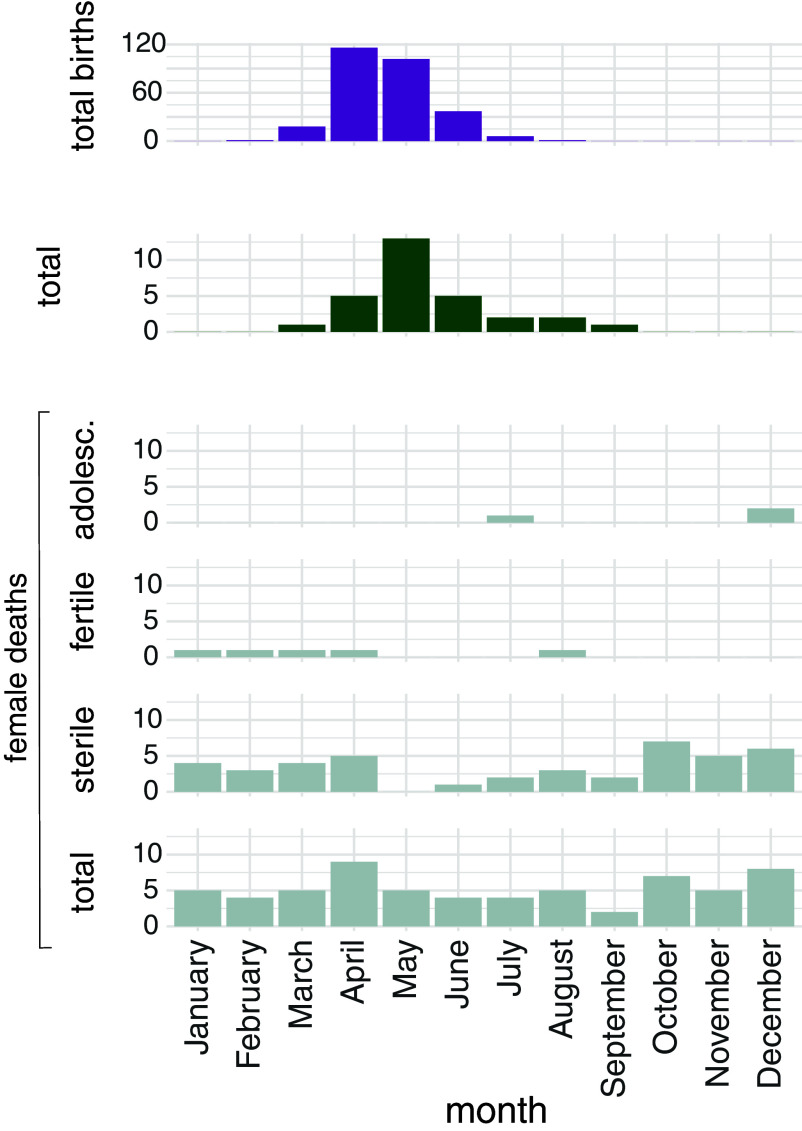
Distributions of birth and death events in the Affenberg Landskron population between 1997 and 2023. The graph displays total births (N = 281), total infant deaths (N = 29), and female deaths per month. Specifically, distributions for female deaths are depicted separately for adolescent females (3.5 to 4.5 y, N = 3), fertile females (>4.5 y; N = 5, excluding two cases with only the year of death reported), and sterile females (>4.5 y, N = 42). The category of immature females (1 to 3.5 y) is omitted due to only having the year of death reported for the sole recorded death in this group. The histogram at the bottom represents the total female deaths (N = 63), the sum of the three groups above including infant female deaths (N = 13). Both births and infant deaths peak during the birth season in April–May. In contrast, the mortality of females at reproductive age does not show such a pattern.

In total, 281 infants were born by 112 females during the observation period (143 female infants, 132 male infants, six of unknown sex). In accordance with Pflüger et al. (42), who reported on all births in this population up to 2019, the birth season ranged from mid-March to July, with most births occurring in April–May (Fig. 2).

No births occurred outside March–July in the additional four years (2020–2023) included in the present study. On average, the age of females at first birth was 4.95 y (SD = 0.83 y). The youngest first-time mother was 3.03 y old, and the oldest first-time mother was 7.85 y. Most females at the Affenberg gave birth to their first offspring between four and five years of age. The mean age at sterilization of all females in our population was 8.57 (SD = 3.03 y, N = 90, including 19 females of the founder group).

### Maternal Mortality.

The mean survival time after giving birth was 9.49 y (SD = 7.20 y; range: 0.31 y to 21.77 y; N = 44). None of the female deaths in this population occurred within 42 d after giving birth. The majority (N = 42) of female deaths occurred among sexually mature sterile females (Fig. 2). We observed seven deaths of sexually mature fertile females (N = 5 with known month of death), three deaths of adolescent females, and only one death of an immature female (unknown month of death). The mortality of the fertile females was not elevated during the birth season (March–July) (Fig. 2). Three females (one sexually mature adolescent and two sexually mature fertile females) died during the birth season. The cause of death was unknown in all three cases, implying that pregnancy cannot be excluded. However, in none of these cases, an infant was found and no signs of birth, which are always present after a birth event, were detected (no bloody thighs, no slimmed appearance, etc.). One of the sexually mature fertile females died on the third of March, making a birth- or labor-related cause of death highly unlikely since all infants were born from mid-March onward (except for one unusual birth in February). The death of the sexually mature adolescent female at the end of July is also unlikely to be associated with pregnancy, labor, or birth because only one female in our entire sample gave birth at an age ≤ 3.5 y. Together, this evidence implies that births did not cause any noticeably higher mortality risk in our study population.

**Fig. 3.**
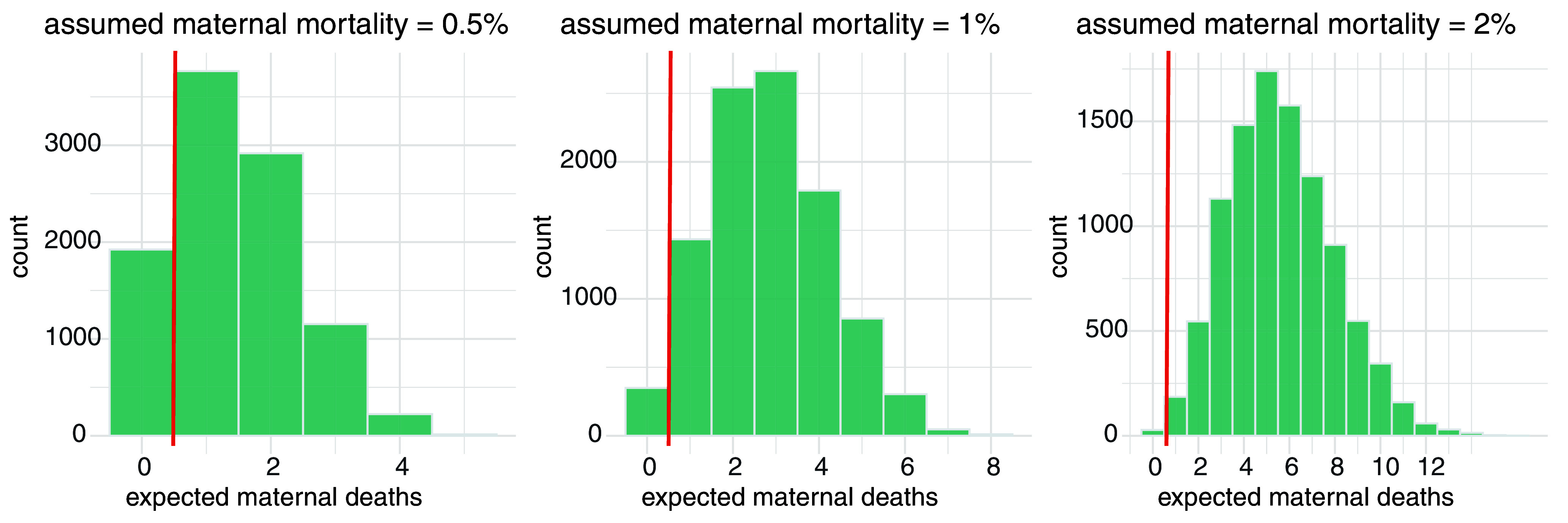
Permutation tests for maternal mortality in macaques. The histograms illustrate the expected maternal deaths at birth, assuming maternal mortality rates of 0.5%, 1%, and 2%, respectively. Each test involved drawing 10,000 random samples, each with a size of N = 281 (the number of births in our macaque data), from a population of 1,000 births, without replacement. The probabilities of obtaining zero maternal deaths by chance in the three tests are *P* = 0.1923, *P* = 0.0350, and *P* = 0.0028, respectively. The probability value corresponds to the area of the green bar at 0, to the left of the red vertical line, relative to the total green area in each plot.

We performed permutation tests to assess whether maternal mortality based on our sample of macaque births can be statistically distinguished from estimates for maternal mortality in human populations (Fig. 3).

The tests determined whether the result of zero maternal deaths in our sample could be due to chance. We calculated the expected number of maternal deaths at birth when sampling from a population with a maternal mortality rate of 0.5%, 1%, or 2%, respectively. These values represent the estimates for human maternal mortality across various populations. We drew 10,000 random samples of size N = 281 (number of births in our macaque data) from a population of 1,000 births with replacement. We then assessed the resulting distribution of maternal deaths in these samples (Fig. 3). The results indicate that a maternal mortality of zero at birth for the 281 births in our data is highly unlikely to occur by chance for a maternal mortality of 1% or 2% (*P* = 0.036 for 1%, *P* = 0.003 for 2%, but *P* = 0.19 for a maternal mortality of 0.5%). This implies that the maternal mortality in our macaque population is statistically significantly lower than 1% (Fig. 3) and may therefore be smaller than estimates of human natural maternal mortality.

### Causes of Female Deaths.

Several females (all above one year of age) died or had to be put down by the veterinarian due to severe injuries (heavy wounding, wound infection, N = 4), accidents (blunt trauma, bone fractures or paralysis, N = 7), infections (sepsis, tetanus, lung infection, N = 6), or cancer (N = 3). For the females, who died above 26 y of age, no specific cause of death other than old age was reported, N = 6). For 27 females, the cause of death was unknown.

### Infant Mortality.

In total, 29 infants (infants are individuals younger than one year of age) died before the age of one year (13 females, 10 males, six of unknown sex). Infant mortality was present in the birth season with a peak in May (13 out of 29 infants died in May, see Fig. 2). The majority of infant deaths (N = 25) occurred within the first 30 d of life (neonatal mortality). The neonatal mortality rate in this group was 8.9%.

### Causes of Infant Deaths.

The cause of death was unknown for most infants. Seven infants died because they either were neglected by the mother or were taken by higher-ranked female relatives (e.g., grandmother). For four cases, the birth date matched the death date and stillbirth cannot be excluded (this includes one case where the mother showed clear signs of having given birth, but the infant body was never found).

### Gynecological Pathologies.

During the observation period of this study, one case of pelvic organ prolapse of a multiparous female was observed. The prolapse occurred three days after giving birth to her third offspring. A colpocleisis was performed shortly after diagnosing the prolapse by a veterinarian. The female had a successful surgery and went on to live for an additional 13 y. Since the female was sterilized shortly afterward, we are unable to report to which extent the condition would have affected a later pregnancy. Apart from this one case, no incidents were reported that required medical treatment in pregnant females, during birth, or within 42 d after giving birth.

## Discussion

Although passage through the maternal pelvic inlet of the birth canal is as tight for Japanese macaque fetuses as it is for human fetuses, the fitness consequences in terms of maternal mortality and morbidity risk caused by birth seem to be quite different, as demonstrated by our data. In this study, no single female macaque death could be linked to a birth event, despite 27 y of data on 281 macaque births. If birth-related mortality in macaques were as high as human natural mortality, noticeable mortality should be present in the months where most births occurred as well as in the ensuing months. In a previous study, Pflüger et al. (42) observed an elevation in female mortality at the onset of the birth season for female Japanese macaques, without distinguishing their reproductive status. This pattern was due to deaths of sterilized females and was not present in the group of fertile females, who could give birth (mature but nonsterilized) (Fig. 2). Notably, according to our data, none of the fertile females died within 42 d after giving birth. Accordingly, we found no evidence that the tight feto-pelvic fit in Japanese macaques is associated with an increased maternal mortality risk.

It remains an open question why birth seems to be less risky in macaques compared to humans. We propose three nonmutually exclusive hypotheses that could explain our observations. i) The macaque fetal skull could be as flexible as the human fetal skull. ii) The macaque pelvis and connective tissue could show greater flexibility during birth than in humans. iii) The birth dynamic of macaques could be less constrained than that of humans due to the difference between the two species in their pelvic morphology.

The capability of the fetal skull to mold during childbirth is an important factor for safe vaginal delivery in humans. The unfused sutures of the human fetal skull permit the frontal and parietal bone edges to slide and overlap in the region of the anterior fontanelle during the second stage of labor (43–45), allowing the fetus to pass through the birth canal more easily. In non-human primates, the open cranial sutures, the posterior fontanelle as well as the thin, taped edges of the frontal and parietal bones support the assumption that molding of the fetal head may also be possible (46). Indeed, in a recent study on rhesus macaques (*M. mulatta*), unfused sutures were generally observed, but the degree of fusion, especially in the metopic suture, varied (7). In summary, it seems that the macaque fetal skull may be able to mold but probably not to the same degree as in humans.

In addition, mobility of the maternal pelvis plays an important role during vaginal birth. Pelvic flexibility is influenced mechanically via different birth positions (47, 48) as well as hormonally (49). It has been shown previously that certain nonhuman primates, including *M. mulatta*, *Saimiri* and *Papio* experience a relaxation of their ligaments during labor (46, 50). As a result, the two hip bones separate substantially, both at the pubic symphysis and sacro-iliac joint, leading to an expansion of the birth canal. This may lead to an increase in the cross-sectional area of the birth canal of up to 30% in *Papio* and up to 100% in *Saimiri* (46). In macaques, it is unknown to what extent pelvic ligaments and muscles relax and expand before and during birth. During pregnancy, the hormones estrogen and relaxin soften the pelvic ligaments and thereby lead to increased flexibility of the entire birth canal. Increased flexibility in the pubic symphysis allows for separation of the pubic bones during birth (1, 51). Progesterone and estradiol were also associated with laxity of the human pelvic ligaments (52–54). Shimizu et al. (55) showed that blood concentrations of progesterone, estradiol-17β, and relaxin also increased before birth in Japanese macaques.

The overall pelvic floor anatomy is similar in humans and macaques (56). The pelvic floor is a muscular diaphragm, which carries several abdominal organs, including bladder, urethra, large intestine, rectum, and anus as well as vagina and uterus in women. In both humans and macaques, the pelvic floor must be remodeled after childbirth to maintain the normal reproductive tract function and position (57). Similar to humans, multiparous macaques are also more vulnerable to pelvic organ prolapse (57, 58), a condition where the abdominal organs drop and bulge into or out of the vagina. Because macaques are intermittently bipedal, the gravitational forces causing pressure on the pelvic floor are presumably reduced, compared to humans, who are obligate bipeds, although resting postures such as upright sitting also put strain on the pelvic floor in macaques (59). Indeed, according to the “pelvic floor hypothesis,” the evolution of a wider pelvis in humans might be constrained by pelvic floor stability (1, 60–63). The evolution of a larger birth canal, which would ease birth, is likely selected against as it would cause increased stress and strain acting on the pelvic floor, leading to higher rates of pelvic floor disorders (1, 61–63). In contrast to the relatively thin and flattened *levator ani* in humans, the *levator ani* in macaques is a short and round muscle group (64). Although it is not known to what extent pelvic ligaments and muscles relax in macaques, it has been shown for other nonhuman primates (e.g., squirrel monkeys and baboons) that the relaxation of the pelvic ligaments and its effect on increasing the flexibility of the birth canal is much greater than in humans (46). We therefore suggest that it is likely that macaques also experience a higher degree of pelvic relaxation similar to other nonhuman primates, which might be a crucial factor enabling easier births in macaques. The mechanism behind such a stronger pelvic relaxation could be higher hormone secretion or a higher density of hormonal receptors in the pelvic tissues. Moreover, Japanese macaques like most nonhuman primates integrate habitual squatting in their daily life and may therefore have a more flexible sacroiliac joint than humans. Recent research (65) hypothesized that regular squatting significantly improves flexion at the sacroiliac joint leading to an enlarged pelvic outlet allowing an easier passage of an infant through the birth canal. Additionally, most of the Japanese macaques, who were observed giving birth, squatted, which constitutes another similarity with humans. Most women giving birth naturally also deliberately choose upright positions like squatting since those positions reduce labor discomfort, enable the fetal head to descend more quickly due to gravity, and increase the width of the pelvic outlet (see review in 66).

The interplay between pelvic form, fetal head size, and fetal position is crucial for successful birth in humans and deviations among these coordinated factors can lead to obstructed labor. In the current macaque literature, one case of obstructed labor linked to a narrow maternal pelvis has been described. This case was resolved by Caesarean section (58). In terms of pelvic morphology and birth dynamics, there are important differences between humans and macaques. In humans, the pelvic inlet has its largest diameter in the transverse direction. The sacral promontory protrudes into the birth canal, narrowing the pelvic inlet sagittally. The ischial spines in the pelvic midplane constrain the birth canal transversally and the sacrum, coccyx, and ischial tuberosities limit the space in the birth canal in the pelvic outlet. As a consequence, in humans, the pelvic inlet, midplane, and outlet are all regions of pelvic constraint (62). By contrast, in macaques, the sacrum is elevated in relation to the pubis and not just the pelvic inlet but the entire birth canal is larger sagittally than transversely. Recent data from Kawada et al. (7) and Laudicina and Cartmill (67) confirmed that the midplane and outlet are also regions of pelvic constraint in macaques: The mediolateral diameter of the birth canal is smallest between the ischial spines in macaques, just like in humans, and the anteroposterior diameter of the outlet is also considerably constrained. Nonhuman primates’ birth canals are not cylindrical either (67) and obstructed labor can occur.

These differences in morphology imply that birth dynamics differ between the two species. Stoller (46) observed that internal fetal neck extension leading to face presentations is common in squirrel monkeys and baboons. Nguyen et al. (68) observed that face presentations appear to also be the norm for geladas (*Theropithecus gelada)*. The results of Kawada et al. (7) further indicate that macaque fetuses perform a similar neck extension leading to a face presentation already at the pelvic inlet, but no internal rotation like humans. Field observations from Duboscq et al. (69) support the view that the neck extension and face presentation is maintained from the pelvic inlet to the pelvic outlet in macaques. Although face-presentations seem to be most frequent, Turner et al. (36) and Nakamichi et al. (41) also reported two cases with vertex presentations, indicating a fully flexed fetal head. In humans, face presentations occur very rarely. Furthermore, nonhuman primates do not perform an external rotation of the head like human fetuses do, where the shoulders and the rest of the body need to rotate to pass through the pelvic outlet.

As human ancestors evolved upright walking, the elongated primate pelvis shortened and changed in shape to suffice the new biomechanical requirements of bipedalism. The pelvis evolved into a shortened bony girdle with pronounced hip joints to accommodate large femoral heads able to support the weight of the spine and upper body. The shape change of the pelvic inlet from an anteroposteriorily oval opening in non-human primates to a mediolaterally oval opening in hominids was what made the complex rotational human birth pattern necessary (61, 70). In summary, human infants have to perform a complex pattern of head rotations, flexion, and extension to successfully pass the bony birth canal, while the nonhuman primate birth dynamic seems simpler (2, 30, 62, 71, 72).

In addition, maternal birth positions can aid the fetal head in entering the birth canal and influence the capacity of the pelvis (73). In particular flexible sacrum positions, i.e., standing, squatting, kneeling, sitting upright, on hands-and-knees, and lateral positions, can expand the pelvic outlet (47, 74) and optimize pelvic capacity (75). Borges et al. (47) estimated based on simulations that the pubic symphysis can widen to a maximum of 3.2 mm and the maximum coccyx rotation is 15.7° in humans. The few birth events that were observed in Japanese macaques showed that females moved freely during labor, alternated different birth positions, and used flexible sacrum positions to give birth (36). Their freedom of movement may allow Japanese macaques to respond better to the requirements of the different stages of labor and find an optimal alternation between birth positions to minimize the risk of obstructed labor.

This study demonstrates that despite a narrow human-like feto-pelvic fit, Japanese macaques appear to have a maternal mortality risk that may be smaller than humans. Furthermore, this study could inspire future research to better understand how freedom of movement and undisturbed physiological labor and birth can lead to more individualized and less invasive maternal care in humans.

## Materials and Methods

Records on birth and death dates, including cause of death and veterinary treatments, were recorded by staff members of the Affenberg Landskron (Affenberg Zoobetriebsgesellschaft mbH, Austria) since the arrival of the group in 1996. The first birth and first death were recorded in 1997. Birth and death records used in the current study covered the years 1997 to 2023. For females born in Japan (females of the founder group), only the year of birth was recorded but the day and month of birth remained unknown. Due to this missing information, founder group females (23 females of which 21 died in the study period) were assigned the birth date of January 1st in the respective year of their birth. For further details on population demographics and the facility, see Pflüger et al. (42).

No prophylactic treatments and no assistance during birth were performed in the Affenberg population to prevent or resolve birth complications or pregnancy-related difficulties. In general, the animals received treatment only in case of life-threatening injuries or acute infections. Apart from one case (prolapse, see above), there were no incidents requiring treatment during pregnancy, birth, or within 42 d after giving birth in the study period. To avoid overpopulation, selective birth control via tubal ligation was performed at Affenberg since 2000. Depending on female kinship and the size of matrilines, females were sterilized after giving birth at least once. Particular attention was paid to maintaining sexually intact females per matriline to protect each female lineage from extinction. In general, female Japanese macaques reach sexual maturity at 3.5 y (76). In the present study, we defined five different female age groups [0: infant (0–1 y); 1: immature (1 y to 3.5 y); 2: adolescent (3.5 y to 4.5 y); 3: sexually mature fertile (>4.5 y); 4: sexually mature sterile (>4.5 y and sterilized)].

For this study, we focused on female mortality and especially on maternal mortality. Maternal mortality is defined by the WHO as death that occurs during pregnancy or within 42 d of termination of pregnancy (17), irrespective of the duration and site of the pregnancy, from any known cause related to or aggravated by the pregnancy, but not from accidental or incidental causes (18). In this study, we applied the WHO definition, with the restriction that deaths during early pregnancy cannot be externally recognized in Japanese macaques. We counted deaths during expected, visible pregnancies as maternal deaths (sexually mature individuals, significant weight increase, not sterilized, no birth in the previous year) and deaths that occurred within the first 42 d postpartum as maternal deaths. Despite two outliers born in February and August (see 42), all other births occurred between March and July.

For 53 of the 66 female individuals (female infants included) that died between 1997 and 2023, the dates of death (day, month, year) were known since they either passed away while receiving veterinary care or they were discovered in the enclosure soon after they passed away. For one individual, we determined the date of death (day, month, year) based on the last date of veterinary care. For five females, we recorded the date when they were last seen as the date of death (day, month, year N = 3; month, year N = 2). For four females, we used the date when their corpse was found as the date of death (day, month, year N = 3; month, year N = 1). Three females (N = 2 sexually mature fertile; N = 1 immature) had to be excluded from the month of death statistic (Fig. 2) since the Affenberg file mentioned only the year of their death. None of these three excluded females had given birth before they died.

In the current study, we also reported deaths of male infants (N = 10) and infants of unknown sex (N = 6). All infant bodies (in total N = 29) were found shortly after death except for one. In this case, the mother showed clear signs of having given birth (i.e., slimmer appearance, bloody thighs, typical gait) but the infant’s body was never found.

The statistical analysis was performed in R (R Core Team, 2022).

## Data Availability

The data and code for this study are openly available online in an OSF repository (77).
